# RNF41 interacts with the VPS52 subunit of the GARP and EARP complexes

**DOI:** 10.1371/journal.pone.0178132

**Published:** 2017-05-22

**Authors:** Delphine Masschaele, Leentje De Ceuninck, Joris Wauman, Dieter Defever, Frank Stenner, Sam Lievens, Frank Peelman, Jan Tavernier

**Affiliations:** 1 Cytokine Receptor Laboratory, Department of Biochemistry, Faculty of Medicine and Health Sciences, Ghent University, Ghent, Belgium; 2 VIB Medical Biotechnology Center, VIB, Ghent, Belgium; 3 Department of Oncology, University Hospital Basel, University of Basel, Basel, Switzerland; Griffith University, AUSTRALIA

## Abstract

RNF41 (Ring Finger Protein 41) is an E3 ubiquitin ligase involved in the intracellular sorting and function of a diverse set of substrates. Next to BRUCE and Parkin, RNF41 can directly ubiquitinate ErbB3, IL-3, EPO and RARα receptors or downstream signaling molecules such as Myd88, TBK1 and USP8. In this way it can regulate receptor signaling and routing. To further elucidate the molecular mechanism behind the role of RNF41 in intracellular transport we performed an Array MAPPIT (Mammalian Protein-Protein Interaction Trap) screen using an extensive set of proteins derived from the human ORFeome collection. This paper describes the identification of VPS52, a subunit of the GARP (Golgi-Associated Retrograde Protein) and the EARP (Endosome-Associated Recycling Protein) complexes, as a novel interaction partner of RNF41. Through interaction via their coiled coil domains, RNF41 ubiquitinates and relocates VPS52 away from VPS53, a common subunit of the GARP and EARP complexes, towards RNF41 bodies.

## Introduction

Eukaryotic cells contain various types of organelles involved in the transport of proteins and lipids. Forward trafficking of cargo in the secretory pathway is counterbalanced by retrograde transport in which resident proteins and transport machinery components are transported back to their original compartment [1–3]. In the endocytic pathway, molecules are internalized at the plasma membrane and delivered to the early endosomes. These early or ‘sorting’ endosomes segregate cargo coming from the plasma membrane into different membrane subdomains. Certain proteins are targeted for lysosomal degradation through the late endosome, while other cargo bypass this step and undergo retrograde transport to the Golgi network, or recycle back to the plasma membrane directly from the endosomes or via the recycling endosomes [4–8]. Biosynthetic, endocytic and retrograde trafficking are highly regulated by the coordinated actions of tethering factors, SNAREs (Soluble N-Ethylmaleimide-Sensitive Factor Attachment Protein Receptors) and small G proteins from the Rab and Arl (Arf-like) family in order to maintain homeostasis of each organelle [9,10].

A key regulatory mechanism of intracellular transport is ubiquitination, which acts as a signal for internalization and sorting of receptors and adaptors [11–14]. The ESCRTs (Endosomal Sorting Complex Required for Transport) further mediate the sorting of ubiquitinated cargo into ILVs (intraluminal vesicles) of MVBs (multivesicular bodies) destined for lysosomal degradation [15,16]. Many E3 ligases have been identified to play a role in endocytosis and lysosomal sorting of membrane proteins. RING (really interesting new gene) finger E3 ligases act as a scaffold to coordinate ubiquitin transfer from an ubiquitin-conjugating E2 enzyme recruited by their RING domain to a specific substrate associated with their substrate binding domain. We previously reported that RNF41 (RING finger protein 41 or Nrdp1 (neuregulin receptor degradation protein-1) or FLRF (fetal liver ring finger) in mouse) controls the sorting and processing of JAK2-associated cytokine receptors including the LR (leptin receptor), LIFR (leukaemia inhibitory factor receptor) and IL6R (interleukin 6 receptor). RNF41 blocks lysosomal sorting and simultaneously enhances ectodomain shedding of these receptors by members of the ADAM (a disintegrin and metalloproteinase) family [17]. It does so by ubiquitinating, destabilizing and relocalizing the deubiquitinase USP8 (ubiquitin-specific protease 8). This leads to a destabilized ESCRT-0 complex, resulting in the rerouting of receptors from the lysosomal degradation pathway to compartments for ectodomain shedding [18]. RNF41 has been implicated in other signaling pathways; it directly ubiquitinates and subsequently downregulates the RARα (retinoic acid receptor alpha), the IL-3 (interleukin-3), EPO (erythropoietin) and ErbB3 receptors, independent of their respective ligands [19–22]. Moreover, RNF41 was reported to differentially regulate Myd88- and TRIF-dependent pathways of TLR4 (Toll-like receptor 4) by ubiquitinating adaptor proteins Myd88 (poly-lys48) and TBK1 (poly-lys63) respectively [23].

To further elucidate the function of RNF41 in intracellular transport we screened a human ORFeome collection containing around 8500 open reading frames in search of novel interaction partners of RNF41 using Array MAPPIT, a high-throughput mammalian two-hybrid screening method [24]. One newly identified RNF41 interaction partner was VPS52 (vacuolar protein sorting 52) which is a key component of two spatially distinct multisubunit tethering complexes, the GARP (Golgi-Associated Retrograde Protein) and EARP (Endosome-Associated Recycling Protein) complexes. The GARP complex is mainly located at the TGN (Trans Golgi Network) and consists of VPS52 together with Ang2, VPS53 and VPS54 [25–27]. This complex functions as a tethering factor for retrograde transport of cargo from endosomes to the TGN. As such, it participates in the delivery of internalized STxB (Shiga toxin B-subunit) and in the retrieval of the M6PR (mannose-6-phosphate receptor), the TGN-resident protein TGN46 and certain SNAREs to the TGN [25,28,29]. The GARP complex is also required for sphingolipid homeostasis and for post-Golgi anterograde transport of GPI-anchored and transmembrane proteins [30,31]. In the EARP complex, VPS54 is substituted by Syndetin while the other subunits are shared with the GARP complex, giving rise to a new tethering complex that associates with recycling endosomes and promotes the recycling of internalized transferrin receptor to the plasma membrane [32].

In this paper we show that RNF41 and VPS52 interact with each other via their CC (coiled coil) domains and demonstrate that RNF41 is able to ubiquitinate and relocate VPS52 from its subcellular location.

## Results

### VPS52 is a novel interaction partner of RNF41

**Fig 1:**
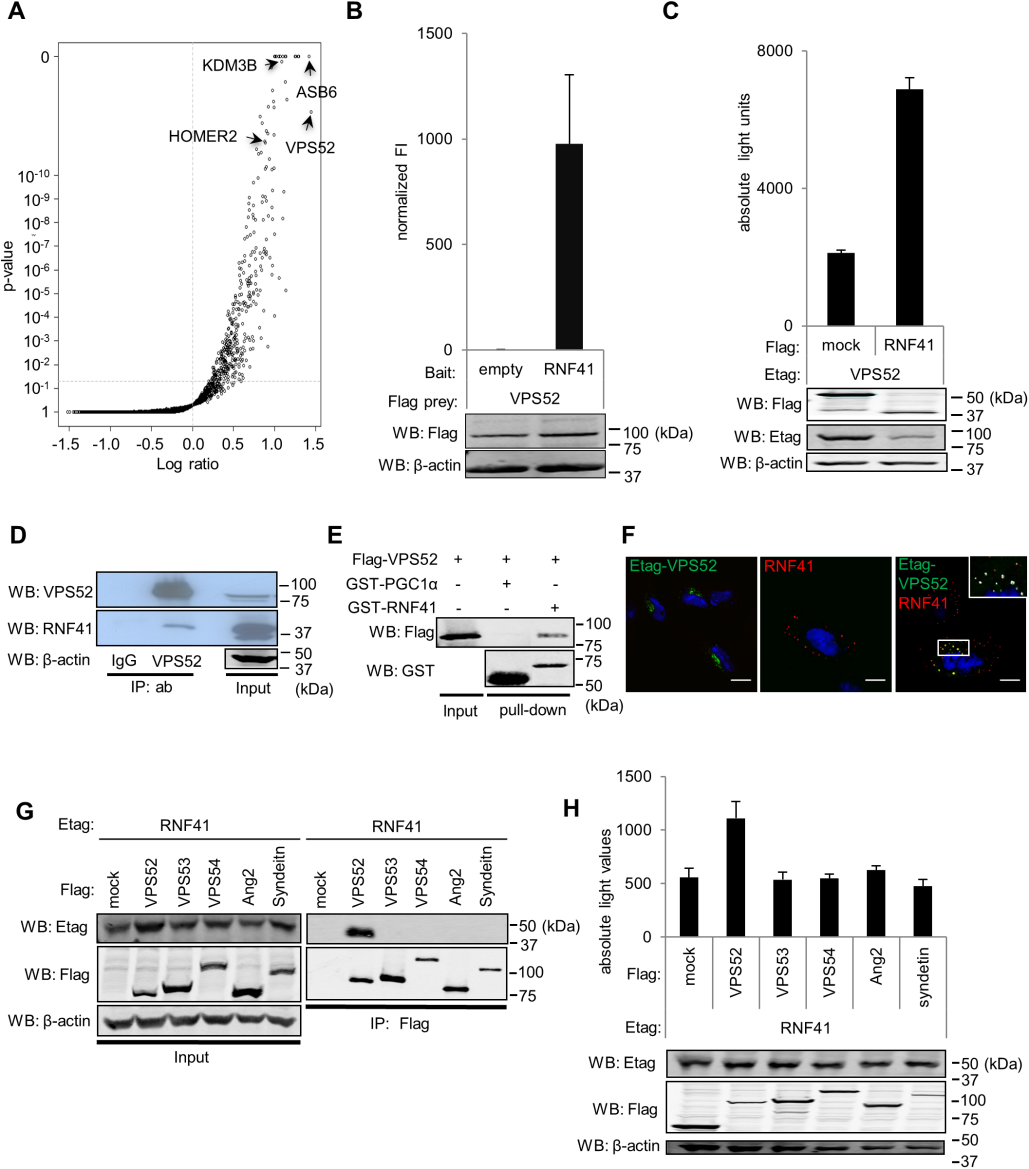
VPS52 is a novel interaction partner of RNF41. **(A)** Array MAPPIT screen result of the RNF41 C34S/H36Q bait against a library covering up to 8,500 preys shown as a volcano plot of the log ratio of normalized MAPPIT luciferase activity versus *P* value. **(B)** MAPPIT analysis of HEK293T cells transiently cotransfected with a vector encoding an RNF41 or empty bait, a VPS52 prey plasmid and the pXP2d2-rPAP1-luciferase reporter plasmid, followed by 24 hours Epo stimulation or left untreated. The luciferase signal is expressed as fold induction (stimulated/non stimulated), relative to the signal of a control JAK2 binding prey ± s.d. of triplicate measurements. Western blotting verified Flag-tagged prey expression and β-actin levels (loading control). **(C)** AlphaScreen analysis of HEK293T cells transiently cotransfected with plasmids encoding E-tagged VPS52 and Flag-tagged RNF41 or SV40 large T antigen (SVT) as an irrelevant protein (mock). After 48 hours, cells were lysed and protein interactions were detected with the AlphaScreen FLAG^TM^ (M2) detection kit (PerkinElmer Life Sciences) by generation of a luminescent signal, displayed as absolute light units. Western blotting shows E and Flag-tagged protein expression and a β-actin loading control. **(D)** Co-immunoprecipitation of endogenous VPS52 and RNF41. HEK293T lysates were immunoprecipitated with anti-VPS52 or a control normal rabbit IgG. The immune complexes (first and second lane) and the input (third lane) were analyzed by immunoblotting with an antibody specific to RNF41 (middle panel). Anti-actin was used as a loading control **(E)** GST-pulldown of *Escherichia coli* BL21(DE3) cell lysates expressing GST-PGC1α (negative control, lane 2) or GST-RNF41 (lane 3) using glutathione sepharose beads and incubated with in vitro transcribed and translated Flag-tagged VPS52. Western blotting using anti-Flag and anti-GST antibodies reveals bead-bound protein complexes and expression of Flag-tagged VPS52 (lane 1). **(F)** Confocal microscopy of HeLa cells transfected with plasmids encoding either E-tagged VPS52 (left panel) or untagged RNF41 (middle panel), together with soluble IL5Rα plasmid as a control, or plasmids encoding E-tagged VPS52 and RNF41 (right panel). Cells were fixed and stained with DAPI (nuclei staining, blue), anti-Etag (secondary Alexa Fluor 488, green) and anti-RNF41 (secondary Alexa Fluor 568, red) antibodies. The inset shows a magnification of the boxed area. The white overlay represents the intersect between RNF41 and VPS52 with a threshold set on standard deviation using Volocity 6.3 software (Perkin Elmer, Waltham, MA, USA). Scale bar, 10μm. n = 3 or more for all performed experiments. **(G)** Co-immunoprecipitation analysis of HEK293T cells transiently cotransfected with plasmids encoding E-tagged RNF41 and Flag-tagged GARP/EARP subunits or soluble IL5Rα. Anti-Flag immunoprecipitates (right panel) and lysates (left panel) were visualized with anti-Flag, anti-Etag and anti-actin. **(H)** AlphaScreen analysis of HEK293T cells transiently cotransfected with plasmids encoding E-tagged RNF41 and Flag-tagged GARP/EARP subunits or SVT.

Array MAPPIT, a high throughput two-hybrid screening method developed in our lab, was used to identify new interaction partners of RNF41 [24]. This method is based on complementation of the JAK-STAT signaling pathway of type I cytokine receptors and allows the detection of protein-protein interactions in intact mammalian cells [33]. Screening was carried out with the RNF41 mutant C34S/H36Q bait, which lacks the ability to recruit an E2 conjugating enzyme. This prevents possible RNF41-dependent ubiquitination and degradation of interacting preys expressed in the system. The RNF41 mutant bait was screened against a human ORFeome collection that covered up to 8.500 preys (list available upon request). VPS52 appeared as one of the top ranked interaction partners of RNF41 amongst other known interaction partners like KDM3B, HOMER2 and ASB6 [34, 35] (Fig 1A). Binary MAPPIT retests confirmed that VPS52 specifically interacted with RNF41 and not with the bait receptor backbone (Fig 1B). AlphaScreen analysis further validated that E-tagged VPS52 specifically interacted with co-expressed Flag-tagged RNF41 in HEK293T cells, and not with a Flag-tagged mock construct, even though E-tagged VPS52 levels in the AlphaScreen lysate fraction were reduced upon RNF41 co-transfection (Fig 1C). Moreover, endogenous RNF41 could be precipitated using an antibody against endogenous VPS52 in HEK293T cells (Fig 1D) and *in vitro* GST-pull down analysis confirmed a direct interaction between VPS52 and RNF41 (Fig 1E). Confocal microscopy of transfected HeLa cells showed a perinuclear localization of VPS52 reminiscent of the trans-Golgi network, while RNF41 localized to punctate cytoplasmic structures. Upon co-transfection, RNF41 and E-tagged VPS52 colocalized in RNF41-positive structures, which we will further refer to as RNF41 bodies(Fig 1F). Of note, the amount of ectopically expressed RNF41 used for confocal experiments was similar to, or below endogenous levels of RNF41 (data not shown). Moreover, RNF41 has a potential myristoylation site at its N-terminus, which could account for membrane anchoring [21], and explain the vesicular pattern of RNF41 observed in Fig 1F. For this reason only C-terminal tagged constructs of RNF41 were used. No difference was observed between expression of N- or C-terminally tagged VPS52. Furthermore, co-immunoprecipitation and AlphaScreen data revealed that RNF41 exclusively interacts with VPS52 and not with the other GARP or EARP subunits (Fig 1G–1E).

### L163 in the coiled-coil domain of RNF41 is required for interaction with VPS52 but not for RNF41oligomerization

**Fig 2:**
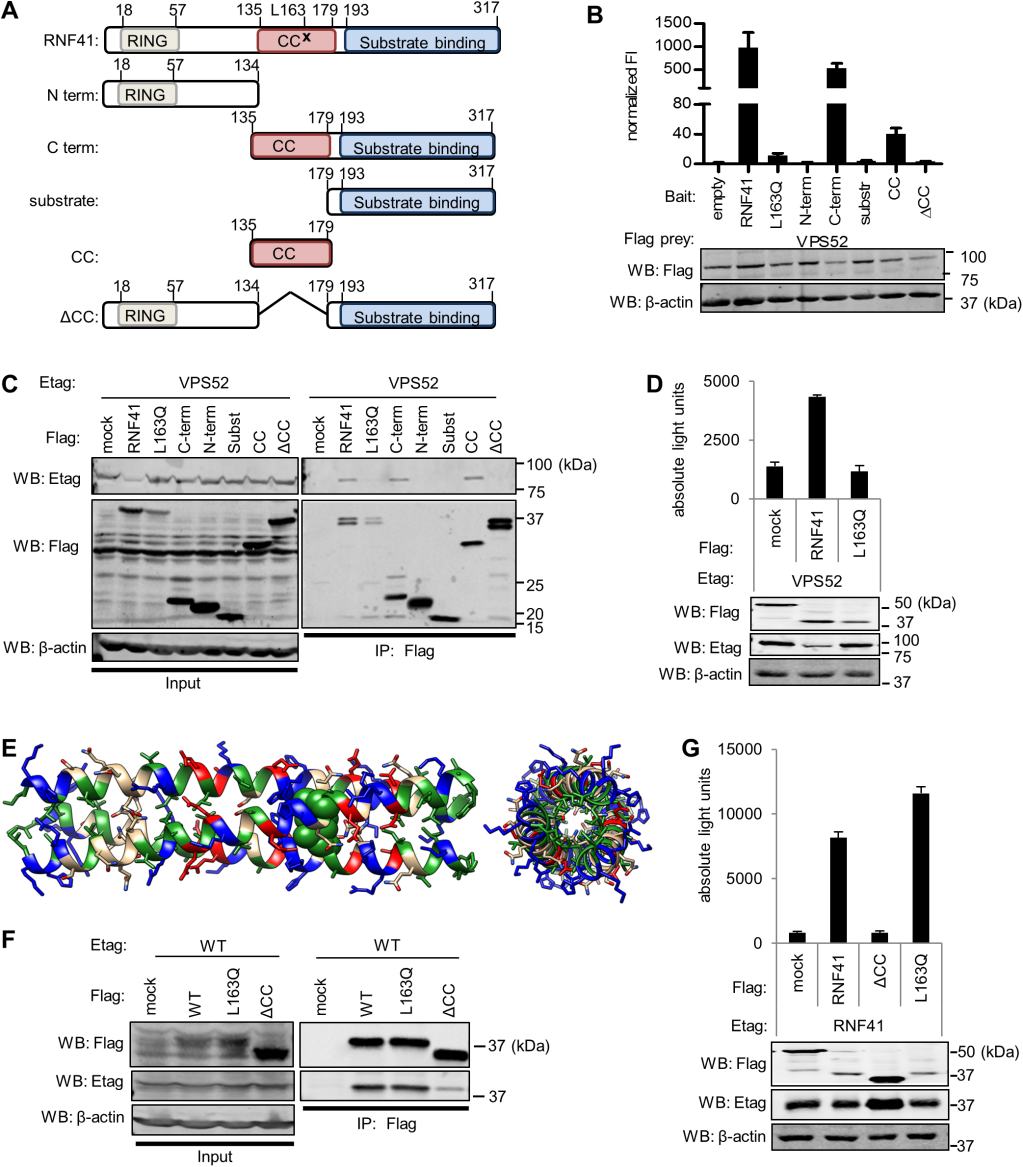
L163 in the RNF41 CC interacts with VPS52 but is not required for RNF41 oligomerization. **(A)** Schematic representation of the amino acid range, L163Q point mutation (indicated with x) and subdomain structure of the full length and truncated RNF41 constructs used to map the VPS52 interaction site. Domain designations: N term, the amino-terminal domain; RING, RING domain; C term, the carboxy-terminal domain; CC, the CC domain; ΔCC, RNF41 construct lacking the CC domain. **(B)** MAPPIT analysis of HEK293T cells transiently co-transfected with a vector encoding a VPS52 prey and an empty, wild type (WT), point mutated or truncated RNF41 bait. **(C)** Co-immunoprecipitation analysis of HEK293T cells transiently co-transfected with a plasmid encoding E-tagged VPS52 and Flag-tagged WT RNF41, L163Q, N-term, C-term, substrate binding domain, RNF41 CC-gp130 (prey construct used to optimize expression), RNF41 ΔCC or soluble IL5Rα (mock). Also Anti-Flag immunoprecipitates (right panel) and lysates (input, left panel) were visualized with anti-Etag, anti-Flag and anti-actin (loading control). **(D)** AlphaScreen analysis of HEK293T cells transiently co-transfected with a vector encoding E-tagged VPS52 and Flag-tagged SVT (negative control), WT RNF41 or L163Q. **(E)** Side (left) and top (right) view of a model of trimeric RNF41 coiled-coils based on the RNF41 sequence using CC-builder and Logicoil. L163 is highlighted as green spheres. **(F)** Co-immunoprecipitation of HEK293T cells transiently co-transfected with E-tagged WT RNF41 and Flag-tagged RNF41, L163Q, RNF41 ΔCC or with Flag-tagged soluble IL5Rα (mock) shows that the L163Q mutation does not hamper RNF41 oligomerization. Anti-Flag immunoprecipitates (right panels) and lysates (left panels) were visualized with anti-Flag and anti-Etag. **(G)** AlphaScreen analysis of HEK293T cells transiently co-transfected with a plasmid encoding E-tagged RNF41 and Flag-tagged SVT, WT RNF41 and L163Q. n = 3 or more for all experiments performed.

To map the interaction of VPS52 on RNF41 we created several truncated forms of RNF41, which were tested in MAPPIT as bait proteins against the VPS52 prey (Fig 2A). The amino-terminal half of RNF41 (AA1-134; N-term) holds a RING domain and the carboxy-terminal half (AA135-317; C-term) consists of a CC domain (AA135-179; CC) and a substrate binding (AA179-317) domain [21]. Only the CC domain and C-terminal RNF41 bait, including the CC domain, interacted with VPS52 prey, demonstrating that the CC domain of RNF41 is necessary for interaction with VPS52 (Fig 2B). To further determine critical RNF41 amino acids involved in the interaction with VPS52, we used an approach that combined random mutagenesis with MAPPIT [36]. Briefly, the MAPPIT RNF41 bait protein was randomly mutated via error prone PCR, covering 113 of a total of 317 amino acids, a mutation yield of about 36% with approximately 26% single mutations. Putative RNF41 mutants were tested against the VPS52 prey or a JAK2-binding prey as a control for expression of the bait receptor. RNF41 mutants were selected based on a single amino acid mutation that strongly decreased the relative MAPPIT signal S1 Table. Mutations at position 163 reoccurred multiple times in the screen and binary MAPPIT retests clearly showed a disruption of the RNF41-VPS52 interaction using the RNF41 L163Q mutant bait (Fig 2B). The L163Q mutation was specific towards VPS52, as interaction between RNF41 and other interaction partners, such as USP8 [37] and ASB6 (manuscript in preparation), was not affected S1 Fig. Significantly, as for the L163Q mutant, all other RNF41 mutants found to disturb the interaction with VPS52 were located in the CC domain of RNF41 S2 Table. This marks VPS52 as the first RNF4 interaction partner that binds to its CC domain. Co-immunoprecipitation (Fig 2C) and AlphaScreen data (Fig 2D), where the RNF41 truncated forms were co-expressed with VPS52 in HEK293T cells, confirmed the MAPPIT results. Of note, the L163Q mutant showed no difference in stability compared to WT RNF41 (data not shown).

The program Logicoil [38], predicted the CC regions of RNF41 to form a trimer, which corresponds to the observations in a recently published paper based on chemical crosslinking and size exclusion chromatography [39] (Fig 2E). The trimeric coiled-coiled core does not consist merely of hydrophobic residues, but also has hydrophilic histidine and asparagine residues. Intermolecular salt bridges outside the core help to stabilize the coil. As RNF41 is able to trimerize via its CC domain, we examined whether the RNF41 L163Q mutation also affects RNF41 oligomerization. Immunoprecipitation and AlphaScreen analysis of transfected HEK293T cells showed that only RNF41ΔCC was able to impair RNF41 oligomerization (Fig 2F and 2G). We conclude that the CC region of RNF41, more specifically residue L163 located in this CC region, is necessary for its interaction with VPS52, while this L163 residue is redundant for RNF41 oligomerization.

### D120, E124 and E127 in the CC domain of VPS52 interact with RNF41 and this interaction decreases RNF41 oligomerization

**Fig 3:**
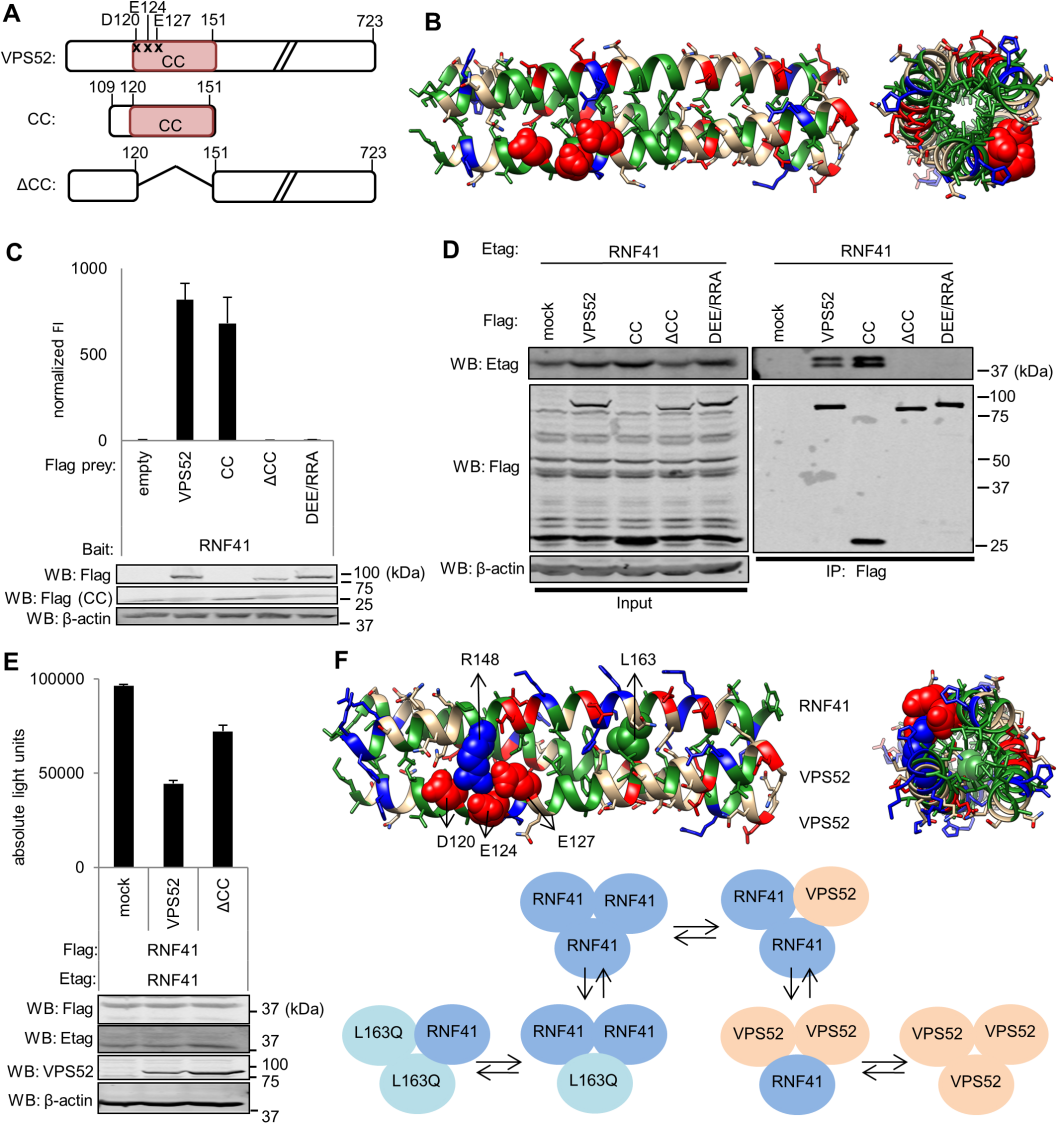
D120, E124 and E127 in the VPS52 CC interact with RNF41 resulting in decreased RNF41 oligomerization. **(A)** Schematic representation of the amino acid range, point mutations (indicated with x) and subdomain structure of the full length and truncated VPS52 constructs used to map the RNF41 interaction site. Domain designations as in Fig 2A. **(B)** Side (left) and top (right) view of a model of trimeric VPS52 coiled-coils based on the VPS52 sequence using CC-builder and Logicoil. D120, E124 and E127 are highlighted as red spheres. **(C)** MAPPIT analysis of HEK293T cells transiently co-transfected with a plasmid encoding an RNF41 bait and an empty; WT; mutated or truncated VPS52 prey. **(D)** Co-immunoprecipitation analysis of HEK293T cells transiently co-transfected with a vector encoding E-tagged RNF41 and Flag-tagged WT VPS52, VPS52CC-gp130 (prey construct used to optimize expression), VPS52ΔCC, VPS52 DEE/RRA or soluble IL5Rα (mock). Anti-Flag immunoprecipitates (right panel) and lysates (left panel) were visualized with anti-Etag, anti-Flag and anti-actin (loading control). **(E)** Ectopic expression of VPS52 hampers RNF41 oligomerization. AlphaScreen analysis of HEK293T cells transiently co-transfected with a plasmid encoding E-tagged and Flag-tagged RNF41 together with WT VPS52, VPS52ΔCC or soluble IL5Rα (mock). Values are means ± s.d from triplicate samples from one of three representative experiments. Data and statistical analysis of biological replicates are shown in S3 Fig. **(F)** Model of RNF41-VPS52 heterotrimeric coiled-coils, consisting of one RNF41 CC (upper coil) and two VPS52 CC (lower coils). R148 and L163 in RNF41 and D120, E124 and E127 in VPS52 are highlighted as blue, green and red spheres respectively. The scheme below depicts the proposed idea of interchangeable monomers between RNF41 and VPS52. n = 3 or more for all experiments.

The Logicoil algorithm also predicted a CC domain in VPS52 between residues 120 and 151. We next created a truncated form of VPS52 without the CC domain (ΔCC), and one existing of only the CC domain (CC) (Fig 3A). We extended the CC domain from residues 109 to 151 as we observed optimal expression of the CC domain using this construct. Modeling of the CC region of VPS52 led to the prediction of a trimeric state with an extensive hydrophobic core (Fig 3B). MAPPIT analysis showed that the CC domain of VPS52 is essential and sufficient for binding with RNF41, as the VPS52 CC prey interacts with the RNF41 bait, while the VPS52 ΔCC prey does not (Fig 3C). Co-immunoprecipitation with ectopically expressed RNF41 and VPS52 mutant or truncated constructs in HEK293T cells verified the MAPPIT results (Fig 3D).

Since RNF41 and VPS52 interact with each other via their respective CC domains (Figs 2B, 2C, 3C and 3D), and RNF41 oligomerizes via its CC domain ([39] and Fig 2F and 2G), we examined whether VPS52 binding to RNF41 interferes with RNF41 oligomerization. VPS52 co-expression impeded the interaction between Flag-tagged and E-tagged RNF41 in AlphaScreen, whereas co-expression of the VPS52ΔCC mutant partially restored this interaction (Fig 3E). As RNF41 homotrimers are prone to auto-ubiquitination and degradation, disruption of RNF41 homotrimers lead to a more stabilized RNF41 expression [39]. We observed this in S2B Fig, where VPS52 co-expression increases RNF41 monomer expression compared to mock- or VPS52ΔCC- transfected cells. This was compensated for in Fig 3E. These results fit a hypothesis where CC RNF41 and VPS52 monomers can be interchangeable to form RNF41-VPS52 heterotrimers. The existence of such RNF41 and VPS52 heterotrimers is supported by molecular modeling that shows high electrostatic compatibility between the helices of RNF41 and VPS52: E153, K160 and K167 of RNF41 respectively face R125, D136 and E143 in VPS52. Furthermore, E165 and E169 in RNF41 are both able to interact with R145 in VPS52, and R148 in RNF41 can interact with D120, E124 and E127 in VPS52 (Fig 3F). Based on this model we selected three negatively charged residues, D120, E124 and E127 in VPS52 (Fig 3A and 3B) which proved to be important for the interaction with RNF41. MAPPIT and co-immunoprecipitation analysis demonstrated that this VPS52 DEE/RRA mutant prey disrupted the interaction with the RNF41 bait (Fig 3C and 3D).

### RNF41 ubiquitinates and relocates VPS52

**Fig 4:**
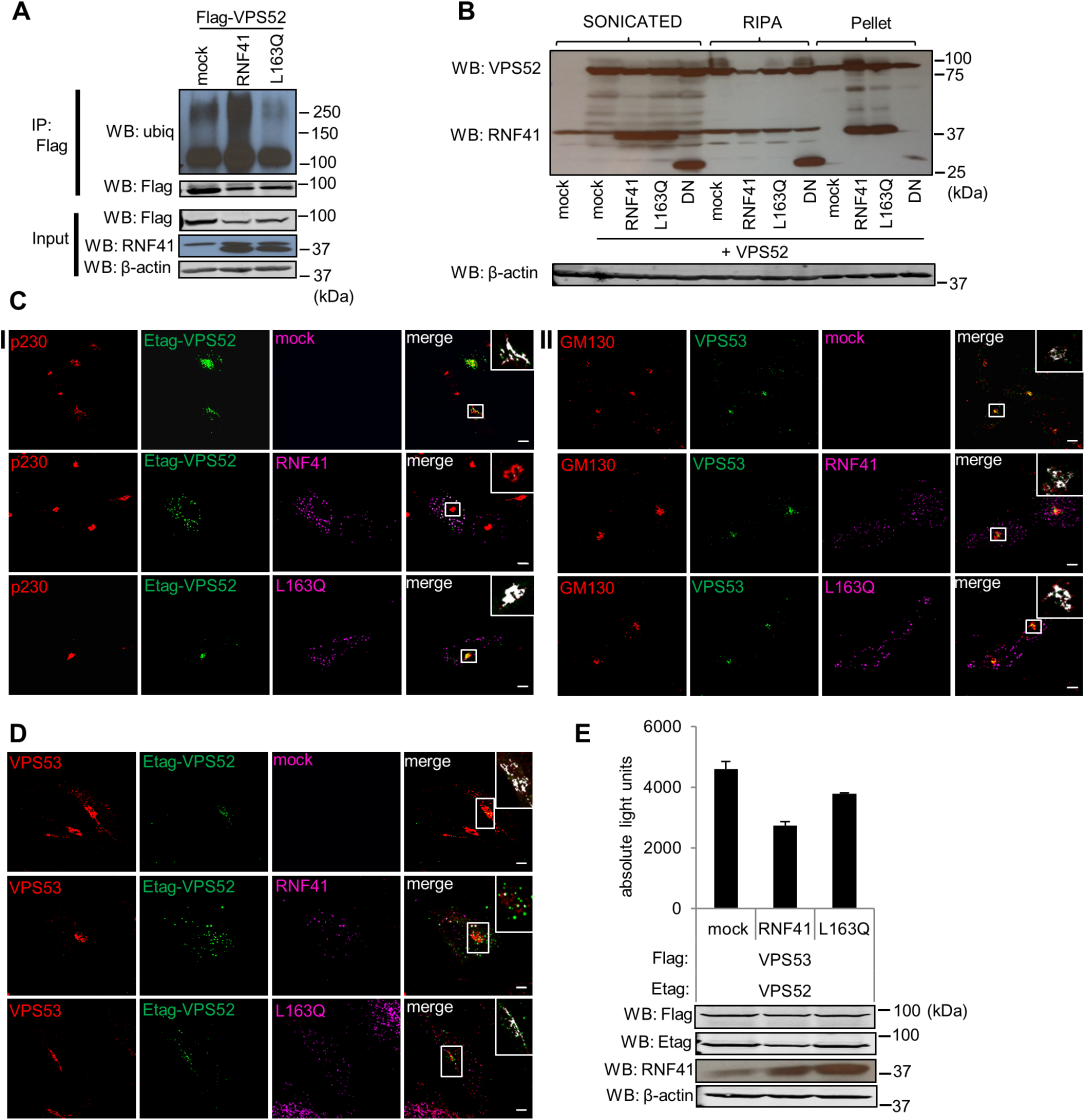
RNF41 ubiquitinates and relocates VPS52. **(A)** RNF41 enhances VPS52 ubiquitination. HEK293T cells co-transfected with a plasmid encoding Flag-tagged VPS52 and untagged RNF41, L163Q or soluble IL5Rα (mock) were incubated overnight with 5mM MG132 to inhibit proteasomal degradation. Flag-VPS52 was immunoprecipitated and ubiquitination was determined by Western Blotting with an anti-ubiquitin antibody (upper panels). Expression and loading controls were visualized using anti-Flag, RNF41 and anti-β-actin antibodies (lower panels). Statistical analysis is shown in S5A Fig. **(B)** RNF41 co-resides with VPS52 in the RIPA insoluble pellet fraction. HEK293T cells transiently co-transfected with a plasmid encoding untagged VPS52 and untagged RNF41, L163Q, DN RNF41 or soluble IL5Rα (mock) were either sonicated in 2x Laemmli buffer (left) or lysed with RIPA buffer (middle). Insoluble RIPA pellets were sonicated in 2x Laemmli buffer (right). Protein levels were detected by Western blotting using anti-VPS52, anti-RNF41 and anti-β-actin (loading control). Statistical analysis is shown in S5B Fig. **(C)** RNF41 relocates VPS52 and not VPS53. (I) Confocal microscopy of HeLa cells transiently transfected with a vector encoding E-tagged VPS52 together with soluble IL5Rα (mock, upper panel), untagged RNF41 (middle panel) or L163Q (lower panel) (right side shows merged images) were fixed and stained with antibodies against Etag (secondary Alexa Fluor 488, green), RNF41 (secondary Alexa Fluor 568, magenta) and Golgi marker p230 (secondary Alexa Fluor 647, red). (II) Same setup as in I, with co-transfection of VPS53 instead of E-tagged VPS52, and the use of anti-VPS53 (secondary Alexa Fluor 488, green) and co-staining with Golgi marker GM130 (secondary Alexa Fluor 647, red). n = 3 or more independent experiments. **(D)** Confocal microscopy and **(E)** AlphaScreen analysis show that Ectopic RNF41 expression disrupts the VPS52-VPS53 interaction. For confocal microscopy, HeLa cells were transiently transfected with a plasmid encoding E-tagged VPS52 and untagged VPS53 together with soluble IL5Rα (mock, upper panel), untagged RNF41 (middle panel) or L163Q (lower panel) (right side shows merged images), fixed and stained with antibodies against Etag (secondary Alexa Fluor 488, green), VPS53 (secondary Alexa Fluor 568, red) and RNF41 (secondary Alexa Fluor 647, magenta). The inset shows a magnification of the boxed area. The white overlay represents the intersect between the golgi marker and E-tagged VPS52 or VPS53 (C) or between E-tagged VPS52 and VPS53 (D) with a threshold set on standard deviation using Volocity 6.3 software. n = 3 or more independent experiments. Scale bar, 10μm. For AlphaScreen analysis, HEK293T cells were transiently co-transfected with a plasmid encoding E-tagged VPS52 and Flag-tagged VPS53 together with WT RNF41, L163Q or soluble IL5Rα (mock). Values are means ± s.d from triplicate samples from one of three representative experiments. Data and statistical analysis of biological replicates are shown in S3 Fig.

Since RNF41 is an E3 ubiquitin ligase, we next assessed ubiquitination of VPS52. In contrast to the L163Q mutant, expression of RNF41 clearly enhanced the endogenous ubiquitination status of Flag-tagged VPS52 (Fig 4A). As increased ubiquitination can result in protein degradation, we analyzed the effect of RNF41 on VPS52 protein expression. Interestingly, different conditions of cell lysis showed markedly contrasting results. Co-expression of RNF41 reduced VPS52 protein levels in the RIPA-soluble fraction (Fig 4B, middle) and caused co-enrichment of RNF41 and VPS52 in the sonicated RIPA-insoluble pellet fraction (Fig 4B, right), while total VPS52 levels remained unchanged when cells were physically disrupted by sonication in Laemmli loading buffer (Fig 4B, left). This indicates that RNF41 causes a partial redistribution of VPS52 to the detergent-insoluble fraction. This effect was lost when using a dominant negative (DN) RNF41, a RNF41 truncated form including residues 109 to 317, thus lacking the RING domain and myristoylation site but retaining the CC domain [20,21], or the L163Q mutant. This indicates that the E3 ligase activity and/or membrane anchoring of RNF41 and its specific interaction with VPS52 are necessary for this RNF41-dependent VPS52 redistribution. Moreover, enrichment of RNF41 and L163Q in the RIPA-insoluble pellet fraction (Fig 4B, right), contrary to DN abundancy in the RIPA-soluble fraction (Fig 4B, middle), indicates that the E3 ligase activity and/or membrane anchoring of RNF41 and not merely its oligomerization are required for RNF41 enrichment in the RIPA-insoluble pellet fraction.

We next used confocal microscopy to determine whether RNF41 altered the intracellular localization of VPS52 in HeLa cells. It is known that VPS52 mainly associates with the TGN, together with the three other subunits of the multimeric GARP complex i.e. VPS53, VPS54 and Ang2 [26,27]. However, VPS52 can also reside at recycling endosomes together with the EARP components, VPS53, Ang2 and Syndetin [32]. To take into account both complexes, we studied the localization of the common GARP and EARP subunits VPS52 and VPS53, solely or together. While ectopically expressed E-tagged VPS52 mainly coincided with the TGN marker p230, RNF41 co-expression caused a redistribution of the large majority of VPS52 to RNF41-bodies with only little overlap between E-tagged VPS52 and p230 remaining (Fig 4C panel I). Localization to the TGN was restored in case of the RNF41 L163Q mutant. No relocalization was observed upon co-transfection of VPS53 and RNF41 or the L163Q mutant, where VPS53 still colocalized with GM130, a Golgi network marker (Fig 4C panel II). Moreover, RNF41 co-expression disrupted the well-established colocalization between VPS53 and E-tagged VPS52 (Fig 4D). Also here, RNF41 reroutes VPS52 away from VPS53, while overexpression of the L163Q mutant does not affect VPS52 localization. AlphaScreen analysis in HEK293T cells ascertained the disruption of the VPS52-VPS53 interaction by RNF41. Ectopic expression of RNF41 compromised the interaction between Flag-tagged VPS53 and E-tagged VPS52, whereas expression of the L163Q mutant did not show this effect (Fig 4E). Altogether, these results show that RNF41 is able to ubiquitinate and relocate VPS52 towards RNF41 bodies, thereby disrupting the interaction with VPS53, a common GARP and EARP subunit.

## Discussion

In this paper, we identify VPS52, a subunit of two distinct tethering complexes, GARP and EARP, as a novel interaction partner of RNF41. VPS52 was identified using the high throughput screening method Array MAPPIT [24] and several other assays confirmed this interaction (Fig 1). Domain mapping revealed that the CC domain of RNF41, located in between the N-terminal RING and C-terminal substrate binding domain [21], is necessary and sufficient for interaction with VPS52 (Fig 2B and 2C). This is remarkable, as all other known RNF41 interaction partners such as USP8, ASB6 and ErbB3 interact with its substrate binding domain [22, 40] and Manuscript in preparation]. A combination of random mutagenesis and MAPPIT [36] of the entire RNF41 bait further enabled the identification of critical amino acids involved in the RNF41-VPS52 interface. In line with the domain mapping results, all residues necessary for the interaction with VPS52 were exclusively located in the CC domain of RNF41. We focused on a RNF41 mutant turning a hydrophobic leucine at position 163 into a polar, non-charged glutamine and found that this single residue is necessary for VPS52 interaction. VPS52 also contains an N-terminal CC domain, which proved to be essential for interaction with RNF41 (Fig 3C and 3D). *In silico* modeling of the CC domains of RNF41 and VPS52 lend further support that L163 was important for VPS52 interaction (Fig 3F) and identified a cluster of three residues in the CC domain of VPS52 that mediated the RNF41 interaction (Fig 3B). Next to the CC domain of RNF41, also membrane association is important for interaction with VPS52 as a RNF41 mutant lacking the putative myristoylation site no longer interacted with VPS52 (unpublished observation). Although not much is known about the protein structure of VPS52 and how it interacts with the GARP and EARP subunits, it has been proposed that the N-terminus holding the CC domain is involved in association with other GARP and possibly also EARP subunits [27,41,42]. Our findings provide the first evidence for the involvement of the RNF41 and VPS52 CC domains in a protein interaction.

Besides mediating heteromeric protein-protein interactions amongst a wide range of proteins, alpha-helical CC domains also have self-associating properties. By modeling its CCs, we predicted a trimeric state of RNF41. This is in line with cross-linking and size exclusion chromatography data indicating that RNF41 can self-assemble into a trimeric complex via its CC domain [39]. Similarly, the CC domains of VPS52 were predicted to form trimers (Figs 2E and 3B). Binding of RNF41 or VPS52 to RNF41 through their respective CC may not be mutually exclusive and CC homology modeling predicted that formation of RNF41-VPS52 heterotrimeric structures might occur (Fig 3F). Intertwining of the alpha-helices of the CC domains of RNF41 and VPS52 might lead to dynamic assembly and disassembly of homo- or heterotrimeric protein complexes. Supporting such model, we observed competition between RNF41-VPS52 interaction and RNF41 toligomerization. VPS52 hampered RNF41 oligomerization while VPS52 ΔCC, unable to interact with RNF41, did not affect RNF41 oligomerization (Fig 3E). Conversely, WT RNF41 and not the L163Q mutant reduced VPS52 oligomerization S2A Fig. RNF41 homotrimers are intrinsically instable due to auto-ubiquitination and subsequent proteasomal degradation. This can be counteracted by disrupting RNF41 homotrimerization through the introduction of an exogenous RNF41 CC domain or by CC domain deletion [39]. Similary, disassembly of RNF41 homotrimers by RNF41-VPS52 complex formation might reduce RNF41 auto-ubiquitination, leading to more stabilized RNF41 expression. In accordance with this, we observed a marked increase in RNF41 monomer expression upon co-expression of VPS52, compared to mock- or VPS52ΔCC- transfected cells S2B Fig. In addition, unlike deletion of the complete CC domain, the RNF41 L163Q mutant did not impair RNF41 oligomerization (Fig 2F and 2G) or auto-ubiquitination (S4 Fig), while both disrupted VPS52 interaction (Fig 2B and 2C). This indicates that this single point mutation in the CC domain is not sufficient to disrupt RNF41 oligomerization and affect auto-ubiquitination. Changes in the oligomerization state of CCs are a widely accepted mechanism that occurs in intracellular transport. SNARE proteins are classical examples of unstructured monomers that can form stable hetero-oligomeric complexes through their CC regions [43]. The MTCs (multisubunit tethering complexes) like the GARP, exocyst and COG (conserved oligomeric Golgi) complex represent another group of multimeric protein complexes in which subunits can be exchanged. These complexes are linked by the presence of predicted short CC regions [44]. Binding of these short CCs is specific but not exclusive as they allow the interaction with different partners at different stages or locations. This is best exemplified by the GARP and EARP complex with the exchange of VPS54 for Syndetin [32] and could potentially be the case for the new RNF41-VPS52 complex.

RNF41 has multiple regulatory functions. Next to ubiquitinating BRUCE, Parkin and USP8 [18,45,46], it is known to downregulate ErbB3 and ErbB4 receptors, as well as several type I cytokine receptors such as the IL-3, EPO, IL-6, LIF and LR [17,19–22]. RNF41 often acts as a sorting signal in the transport of these cargo proteins. It reroutes IL6Rα, LIFRα and LR from the lysosomal degradation pathway to compartments where ectodomain shedding occurs by ubiquitinating and suppressing USP8 [17,18]. Different functions of RNF41 can be ascribed to distinct locations as ER-localized RNF41 is responsible for downregulating steady state levels of signaling competent ErbB3 receptors by routing them to the ERAD pathway [47], while RNF41 present in endosomes can ubiquitinate internalized ErbB3 receptors and as such reroute them away from recycling pathways to the lysosomes for degradation [48]. All these known functions of RNF41 are mediated by its RING domain, and substrates are known to interact with its C-terminal substrate binding domain [18, 21]. Here, we present data implying a crucial role for the RNF41 CC domain in recruiting VPS52. RNF41, unlike the L163Q mutant, ubiquitinated VPS52 (Fig 4A). Moreover, we observed a reduction in VPS52 protein levels upon RNF41 expression in the soluble fraction of RIPA-lysed cells. Next to specific interaction between RNF41 and VPS52, this effect was also dependent on functional E3 ligase activity and/or membrane association as the L163Q mutant and a truncated RNF41 mutant with dominant-negative properties (DN RNF41 [20,21]) did not decrease VPS52 levels. Surprisingly, VPS52 levels were maintained when cells were lysed using sonication, pointing more towards an RNF41-dependent redistribution rather than degradation of VPS52 (Fig 4B). Previous studies showed that the GARP complex largely associates with the TGN, although the presence of vesicles coinciding with late, early and recycling endosomal markers were also reported, while the EARP complex localizes only to recycling endosomes [26, 29, 32, 41, 49]. In our hands, the expression pattern of the shared GARP and EARP subunits VPS52 and VPS53 primarily overlapped with Golgi markers. RNF41 relocated the majority of VPS52 away from the TGN towards RNF41 bodies, while VPS53 remained TGN associated (Fig 4C). This is also reflected in the reduced interaction between VPS52 and VPS53 upon RNF41 expression (Fig 4D–4E). These results are consistent with our data indicating that RNF41 solely interacts with VPS52, and not with the other GARP or EARP subunits (Fig 1G and 1H). It also further supports the observation that both RNF41 and VPS52 were enriched in the insoluble sonicated RIPA pellet fraction (Fig 4B), indicating that RNF41 is able to relocate part of VPS52 towards insoluble compartments. It remains to be determined which ubiquitin-chain linkage is responsible for the observed VPS52 ubiquitination, K63- and/or M1-linked chains that control localization of proteins would be in line with the observed relocalization of VPS52 by RNF41 (Komander et al, 2012). However, we cannot exclude the possibility of indirect VPS52 ubiquitination, since the mode of interaction with VPS52 (via the CC domain) differs from the other known RNF41 substrates (via the substrate binding domain). An alternative hypothesis is that RNF41 first recruits VPS52 through its CC domain, resulting in rerouting followed by ubiquitination at its new subcellular location by RNF41 or potentially another unidentified E3 ubiquitin ligase. It is possible that interactions with its CC region are reserved for proteins that regulate RNF41, as exemplified in literature where certain proteins like NS1, MAGEA1 and MAGEC2, are known to respectively interact with the CC domain of RING finger proteins TRIM25, TRIM31 and TRIM28 thereby inhibiting or enhancing their E3 ligase activity [50–52].

In conclusion, we identified VPS52, a component of the GARP and EARP complexes, as a novel interaction partner of RNF41 and show that they interact via their CC domains. RNF41 ubiquitinates and relocates VPS52 away from VPS53, another shared subunit of the GARP and EARP complexes, towards RNF41-positive structures. The nature of these RNF41 bodies which incorporate VPS52 remains to be fully elucidated.

## Materials and methods

### Plasmids and constructs

The generation of all sequence-verified constructs is shown in S3 Table.

### Array and binary MAPPIT analysis

Array MAPPIT and the preparation of the prey and reporter reverse transfection mixture was previously described [24]. The screened prey collection entails 8.569 full length human ORF preys selected from the human ORFeome collection version 5.1 (http://horfdb.dfci.harvard.edu/hv5). For binary MAPPIT, 1 x 10^4^ HEK293T cells (www.atcc.org, mycoplasma negative) were seeded in a 96-well and transfected with 50 ng of STAT3-dependent pXP2d2-rPAP1-luciferase reporter, 250 ng bait and prey constructs using calcium phosphate. Cells were left untreated or stimulated for 24 hours with human Erythropoietin (5 ng/ml). Luciferase activity from triplicate samples was measured by chemiluminescence in an EnVision plate reader (PerkinElmer) and expressed as fold induction (stimulated/non-stimulated relative light units) relative to the signal generated by a JAK2 binding prey, which corrects for possible varying expression levels of the different baits used. Random mutagenesis of RNF41 bait was performed as described in Uyttendaele *et al*, 2012.

### AlphaScreen

2 x 10^5^ HEK293T cells were seeded in a 6-well and transfected with 1 μg of both E- and Flag-tagged proteins using calcium phosphate. In the condition where E- and Flag tagged RNF41 are co-expressed with VPS52, only 250 ng of RNF41 was transfected. AlphaScreen experiments were performed according to the manufacturer’s protocol (PerkinElmer). 48 h later, cells were lysed in TAP lysis buffer (50 mM Tris–HCl pH 7.5, 125 mM NaCl, 5% glycerol, 0.2% NP40, 1.5 mM MgCl2, 25 mM NaF, 1 mM Na_3_VO_4_ and Complete^TM^ Protease Inhibitor without EDTA Cocktail (Roche)). Lysates were incubated for 2 hours at 4°C with 0.7 mg/ml biotinylated anti-E-tag antibody, subsequently incubated for 1 hour at 4°C with the AlphaScreen FLAG™ (M2) detection kit (PerkinElmer Life Sciences) acceptor beads, and finally for 30 minutes at room temperature with streptavidin donor beads. Samples were measured in triplicate using the EnVision plate reader (PerkinElmer). Expression was analyzed by Western Blotting.

### GST-pulldown

BL21(DE3) cells were transformed with GST-constructs. Protein production was induced by 0.2 mM isopropyl-D-thiogalactoside at A600 of 0.6. The bacteria were cultured overnight at 25°C. After centrifugation at 5000 r.p.m. for 10 minutes, the bacterial pellet was resuspended and sonicated in NETN buffer (20 mM Tris-HCl pH 8; 100 mM NaCl; 6 mM MgCl2; 1 mM EDTA; 0.5% NP40; 1% DTT and Complete^TM^ Protease Inhibitor Cocktail from Roche). The lysate was centrifuged at 12,000 r.p.m. for 10 minutes to obtain the soluble GST proteins in the supernatant. GST proteins were immobilized on Glutathione Sepharose 4B beads (GE Healthcare) for 1 hour at 4°C and washed three times with NETN buffer. Flag-tagged VPS52, expressed using the TNT T7 Quick Coupled Transcription/Translation System (Promega), was incubated overnight with the GST beads. The beads were washed with NETN buffer and Flag-tagged proteins bound to the GST-proteins were eluted by 10 minutes boiling in 2x SDS gel loading buffer and analyzed by Western blotting.

### Western blot analysis

1.5 x 10^5^ HEK293T cells were seeded in a 12-well and co-transfected with 1μg of each construct. Cells were washed with PBS, and lysed in RIPA buffer (50 mM Tris-HCl pH 8.0; 200 mM NaCl; 0.05% SDS; 2 mM EDTA; 1% NP-40; 0.5% deoxycholic acid; 1 mM Na3VO4; 1mM NaF; and Complete^TM^ Protease Inhibitor Cocktail (Roche)), or in 2x SDS gel laemmli buffer (62.5 mM Tris- HCl pH 6.8, 3% SDS, 10% glycerol, 5% β-mercaptoethanol and 0.01% Bromophenol Blue sodium salt) and sonicated using the Bioruptor Plus (Diagenode). After boiling, cell lysates were resolved by SDS-PAGE and transferred to nitrocellulose membranes (Amersham Biosciences). Blots were blocked in Odyssey blocking buffer (LICOR), when using Odyssey infrared imaging (LICOR) or in 5% milk upon ECL (enhanced chemiluminescence) detection. Rabbit anti-VPS52 (1:1500, kind gift from F. Stenner-Liewen) and rabbit anti-RNF41 (1:10.000; Bethyl) were revealed by SuperSignal West Pico Chemiluminescent Substrate (Pierce) using peroxidase-conjugated anti-rabbit antibody (1:10.000, Jackson ImmunoResearch), diluted in milk blocking buffer. Mouse anti-β-actin (1:5000, Sigma) and anti-mouse Dylight 680-conjugated antibody (1:15.000, Pierce) diluted in Odyssey blocking buffer + 0.1% Tween20 was used as a loading control. The following antibodies were used for expression controls in other experiments: anti-Flag rabbit or mouse (1:5000 and 1:10.000, Sigma), anti-E-tag mouse (1: 10.000, Phadia) and anti-GST rabbit (1:5000, Abcam) revealed with anti-rabbit or anti-mouse DyLight 800- or DyLight 680-conjugated antibody (1:15,000, Pierce) diluted in Odyssey blocking buffer +0.1% Tween20. For visualization of the ubiquitin signal we used mouse anti-ubiquitin (VU-1, 1: 1000, LifeSensors), revealed with peroxidase-conjugated anti-mouse antibody (1:10.000, Jackson ImunoResearch), diluted in milk blocking buffer.

### Co-immunoprecipitation analysis

HEK293T cells (1.8 x 10^6^ in a 60 mm petri dish) were transfected with 3μg of each construct for 48 hours and cell extracts were prepared in lysis buffer (50 mM Tris HCl pH 7.5; 125 mM NaCl, 0.2% NP40; 1.5 mM MgCl2; 5% glycerol and Complete^TM^ Protease Inhibitor Cocktail from Roche) followed by two freezing rounds of 10 minutes at -80°C. The lysates were cleared by centrifugation and precleared with sepharose 4B beads (Sigma) followed by incubation overnight at 4°C with 20μl of monoclonal anti-flag M2 agarose beads (Sigma) to precipitate the Flag-tagged proteins. Immunoprecipitates were washed three times with lysisbuffer and eluted from the beads using Flag peptide (200μg/ml, Sigma). After adding 5x SDS gel loading buffer, samples were analyzed by Western blotting. For endogenous Co-IPs, HEK293T cells were lysed and prepared as described above, After preclearing, 1 ml of lysate was incubated overnight with 1.5 μg of rabbit anti-VPS52 or normal rabbit IgG (Santa Cruz Biotechnology). Subsequently, lysates were incubated with protein A sepharose beads (Sigma) for 2 hours at 4°C. The beads were washed three times and resuspended in 2 x SDS gel loading buffer.

### Ubiquitination assay

HEK293T cells (1.8 x 10^6^ in a 60 mm petri dish) were transfected with 6μg of each construct together with 3μg of Flag-tagged VPS52. The next day, cells were washed and treated overnight with 5 mM MG132 in serum-free OPTIMEM medium (Invitrogen). 48h post-transfection, cells were washed with PBS and lysed in 250 μl of SDS lysis buffer (2% SDS, 150 mM NaCl, 10 mM Tris-HCl, pH 8.0, 2 mM sodium orthovanadate, 50 mM sodium fluoride, 10 mM N-ethylmaleimide and Complete^TM^ Protease Inhibitor without EDTA Cocktail from Roche). Lysates were sonicated using the Bioruptor Plus (Diagenode), boiled for 10 minutes and diluted in 2250 μl dilution buffer (10 mM Tris-HCl, pH 8.0, 150 mM NaCl, 2 mM EDTA, 1% Triton X-100) for 30–60 minutes at 4°C under rotation. The lysates were cleared by centrifugation for 30 minutes and precleared with sepharose 4B beads (Sigma) followed by incubation overnight at 4°C with 20μl of monoclonal anti-flag M2 agarose beads (Sigma) to precipitate the Flag-tagged proteins. Immunoprecipitates were washed three times with wash buffer (10 mM Tris-HCl, pH 8.0, 1 M NaCl, 1 mM EDTA, 1% NP-40) and resuspended in 2x SDS gel loading buffer followed by Western blot analysis.

### Confocal microscopy

1.5 x 10^5^ HeLa cells (www.atcc.org, mycoplasma negative) were seeded on No.1.5 glass coverslips (Zeiss) in a 6 well coated with poly-L-lysine (Sigma-Aldrich). The next day, cells were transfected with 100 ng of construct using JetPrime (Polyplus). 24 hours later, cells were rinsed with PBS and fixed for 15 minutes at room temperature in 4% paraformaldehyde. For Shiga toxin B subunit internalization, 24 hours after transfection, cells were incubated with 1μg/ml STxB-Cy3 (kind gift from L. Johannes) for 30 minutes and chased in DMEM for 60 minutes prior to fixation. Cells were washed with 100mM phosphate buffer (100 mM Na_2_HPO_4_; 100 mM NaH_2_PO_4_, pH 7.4), permeabilized and blocked in blocking buffer (20 mM phosphate buffer; 100 mM NaCl; 0.23% Triton X-100 and 10% donkey serum) for 30 minutes. Samples were incubated for 1.5 hours at room temperature with goat anti-Etag (1:2000, Bethyl), mouse anti-RNF41 (1:500, Santa Cruz), rabbit anti-p230 (1:800, Santa Cruz), goat anti-GM130 (1:150, Santa Cruz) or rabbit anti-VPS53 (1:1500, Abcam). After washing in blocking buffer without donkey serum, cells were incubated for 1 hour at room temperature with donkey anti-goat Alexa Fluor 488 or 647, donkey anti-mouse Alexa Fluor 568 or 647 and donkey anti-rabbit Alexa Fluor 647, 488 or 568. Images were acquired using a 60x 1.35 NA objective on an Olympus IX-81 laser scanning confocal microscope. Intersect was determined using a threshold set on standard deviation intensity in the Volocity 6.3 software (Perkin Elmer, Waltham, MA, USA). Noise removal was done with medium filters.

## Supporting information

S1 Table Random mutagenesis combined with MAPPIT, single mutants. Table representing all single RNF41 mutants resulting from the screen. Column 1 and 2 show the positions and mutations in RNF41. Column 3, 4 and 5 represent the relative MAPPIT signals of the preys tested against the RNF41 mutants, with EFH1A, a JAK2 binder, as a positive control. The relative MAPPIT signal was calculated as the median of the normalized MAPPIT value, which is the result of the fold induction of each mutant divided by the median of the fold induction of the six WT controls on that plate.
(XLSX)

S2 Table Random mutagenesis combined with MAPPIT, specific single mutants. Table representing only the specific single mutants for interaction with VPS52 or ASB6.
(XLSX)

S3 Table Generation and origin of the constructs used in this paper. (XLSX)

S1 Fig L163Q specifically disrupts interaction with VPS52. MAPPIT analysis of HEK293T cells transiently co-transfected with a plasmid encoding an empty, VPS52, ASB6 or USP8 prey together with a RNF41 or L163Q bait.
(TIF)

S2 Fig **(A) Ectopic expression of RNF41 hampers VPS52 oligomerization.** AlphaScreen analysis of HEK293T cells transiently co-transfected with a plasmid encoding an E-tagged and Flag-tagged VPS52 together with untagged WT RNF41, L163Q or sol IL5Rα (mock). Values are means ± s.d from triplicate samples from one of three representative experiments. Data and statistical analysis of biological replicates are shown in S3 Fig. **(B) Ectopic expression of VPS52 hampers RNF41 oligomerization and auto-ubiquitination thereby stabilizing RNF41.** HEK293T cells transiently co-transfected with a plasmid encoding an E-tagged and Flag-tagged RNF41 together with WT VPS52, VPS52ΔCC or sol IL5Rα (mock).
(TIF)

S3 Fig Data and statistical analysis of biological replicates from AlphaScreen experiments in Figs 3E, 4E and S2. One-way ANOVA (randomized block design) showed a marginal significant difference (Fig 3E: P = 0.0983; Fig 4E: P = 0.1106; S2 Fig: P = 0.0693).
(TIF)

S4 Fig L163Q does not influence RNF41 auto-ubiquitination. HEK293T cells co-transfected with pMet7-vectors encoding Flag-tagged RNF41, L163Q, ΔCC or sol IL5Rα (mock), together with HA-ubiquitin were incubated overnight with 5μM MG132 and 25μM chlolorquine to inhibit proteasomal or lysosomal degradation. Flag immunoprecipitation followed by anti-HA staining revealed the ubiquitination state of the RNF41 mutants.
(TIF)

S5 Fig Statistical analysis of biological replicates from the data in Fig 4A and 4B. The Western Blots in Fig 4A and 4B, together with biological replicates, were quantified using Image J. **(A)** The ubiquitination signal was normalized for the amount of immunoprecipitated Flag-tagged VPS52 and compared between the mock, RNF41 and L163Q condition. A one-way ANOVA (randomized block design) showed a significant difference in VPS52 ubiquitination between the RNF41 and mock or L163Q transfected cells (p<0.01). **(B)** For each condition (i.e. mock, RNF41, L163Q and DN ectopic expression) the amount of VPS52 in the soluble RIPA and insoluble pellet fraction was compared to the total amount of VPS52 in the sonicated fraction. These results were subjected to a two-way ANOVA with post-hoc comparison (Bonferroni correction) that showed statistical difference between RNF41 and mock or L163Q (p<0.05) for the RIPA fraction and between RNF41 and mock or L163Q (p<0.001) for the pellet fraction.
(TIF)
