# Supplementary figures and images for: RNF41 interacts with the VPS52 subunit of the GARP and EARP complexes
Source: PLoS One. 2017 May 22;12(5):e0178132. doi: 10.1371/journal.pone.0178132 (PMC5439944; doi:10.1371/journal.pone.0178132)

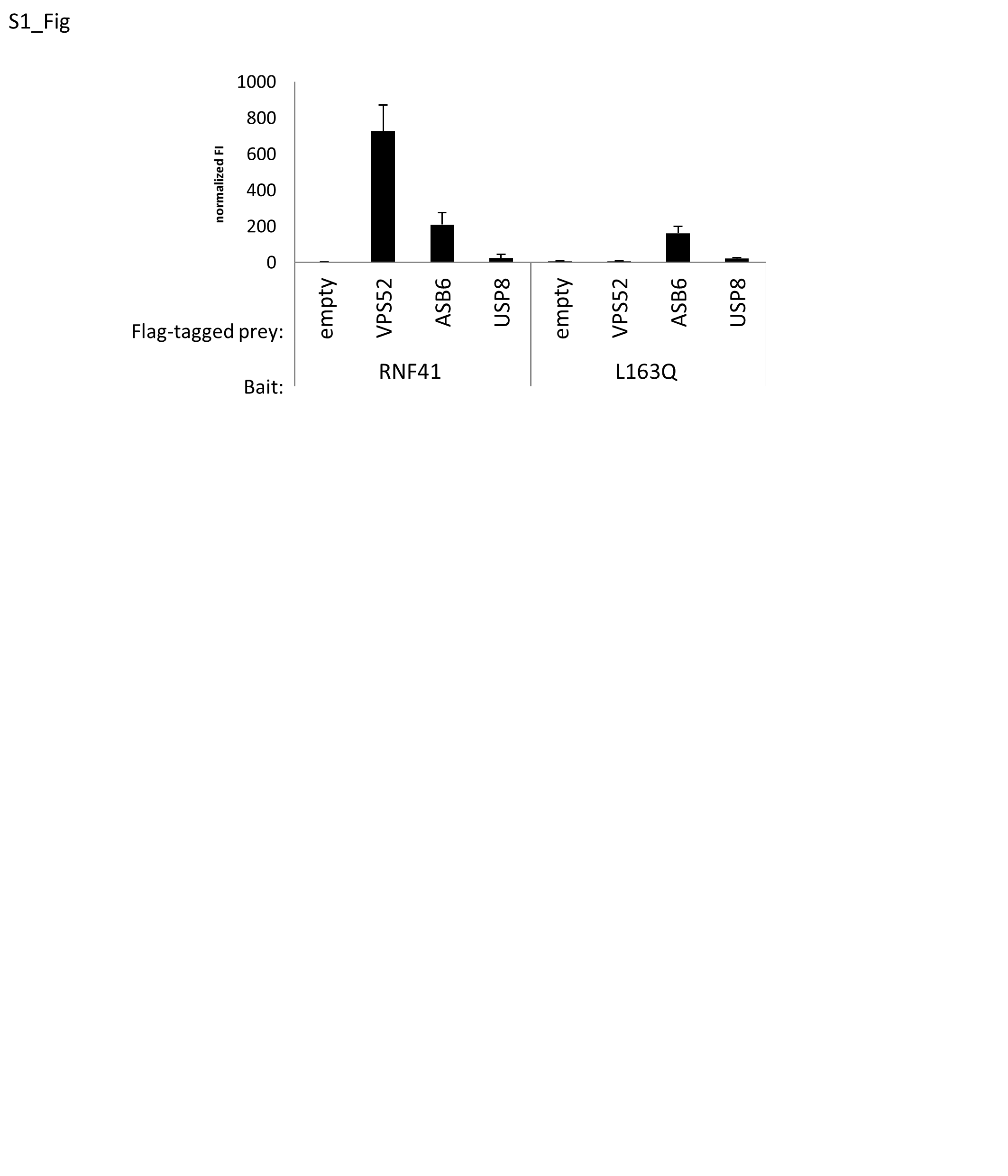

Supplement: S1 Fig — MAPPIT analysis of HEK293T cells transiently co-transfected with a plasmid encoding an empty, VPS52, ASB6 or USP8 prey together with a RNF41 or L163Q bait. (TIF) [file pone.0178132.s004.tif]

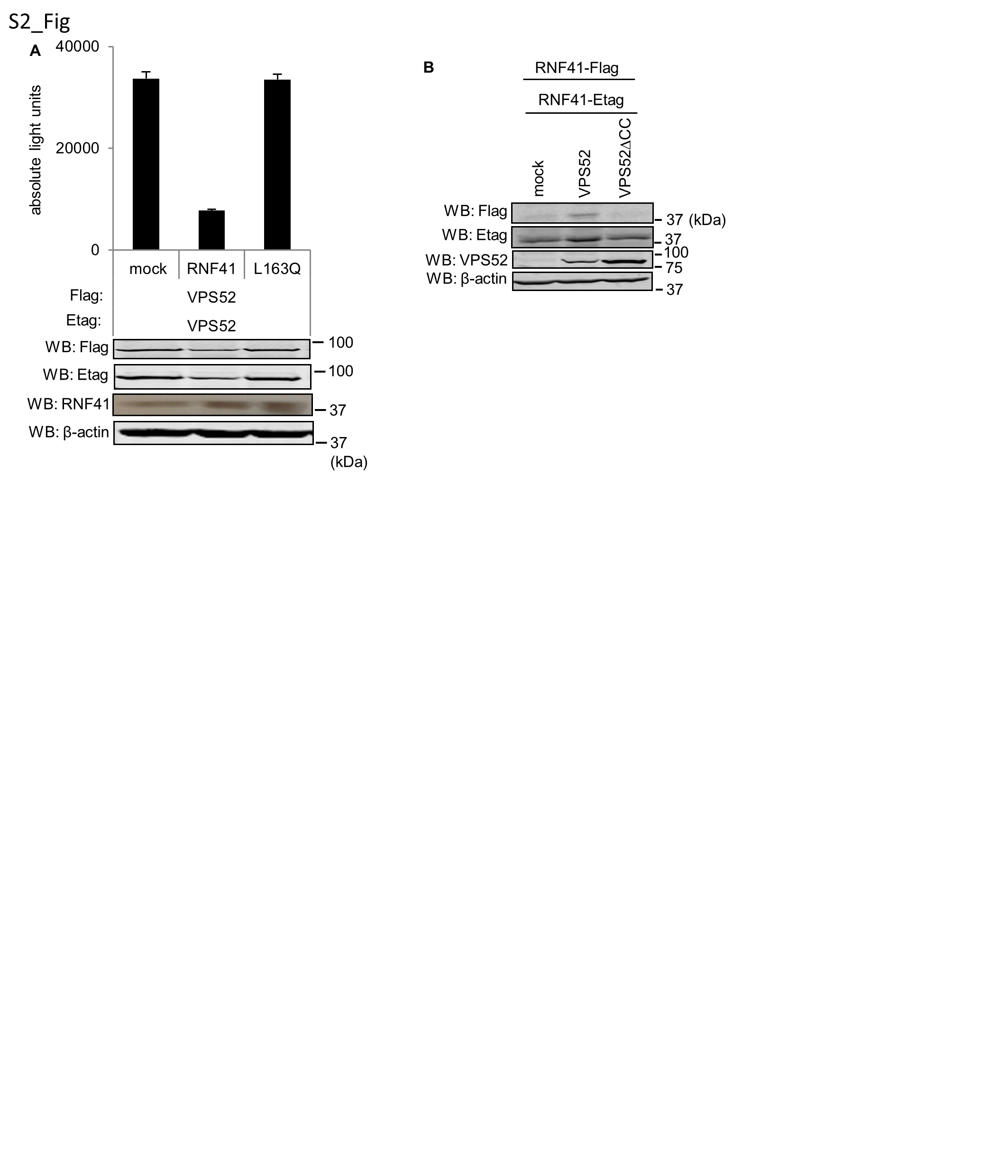

Supplement: S2 Fig — (A) Ectopic expression of RNF41 hampers VPS52 oligomerization. AlphaScreen analysis of HEK293T cells transiently co-transfected with a plasmid encoding an E-tagged and Flag-tagged VPS52 together with untagged WT RNF41, L163Q or sol IL5Rα (mock). Values are means ± s.d from triplicate samples from one of three representative experiments. Data and statistical analysis of biological replicates are shown in S3 Fig. (B) Ectopic expression of VPS52 hampers RNF41 oligomerization and auto-ubiquitination thereby stabilizing RNF41. HEK293T cells transiently co-transfected with a plasmid encoding an E-tagged and Flag-tagged RNF41 together with WT VPS52, VPS52ΔCC or sol IL5Rα (mock). (TIF) [file pone.0178132.s005.tif]

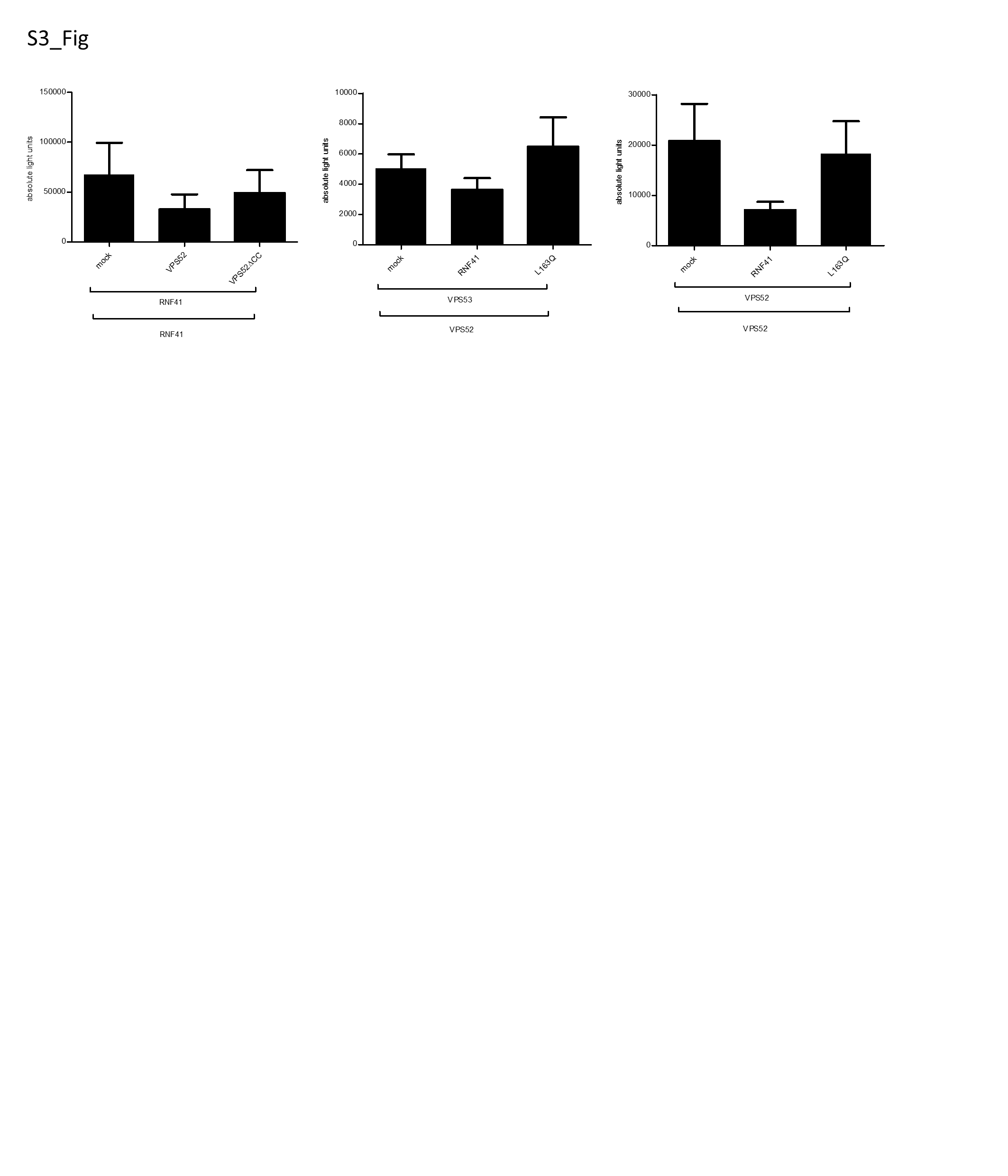

Supplement: S3 Fig — One-way ANOVA (randomized block design) showed a marginal significant difference (Fig 3E: P = 0.0983; Fig 4E: P = 0.1106; S2 Fig: P = 0.0693). (TIF) [file pone.0178132.s006.tif]

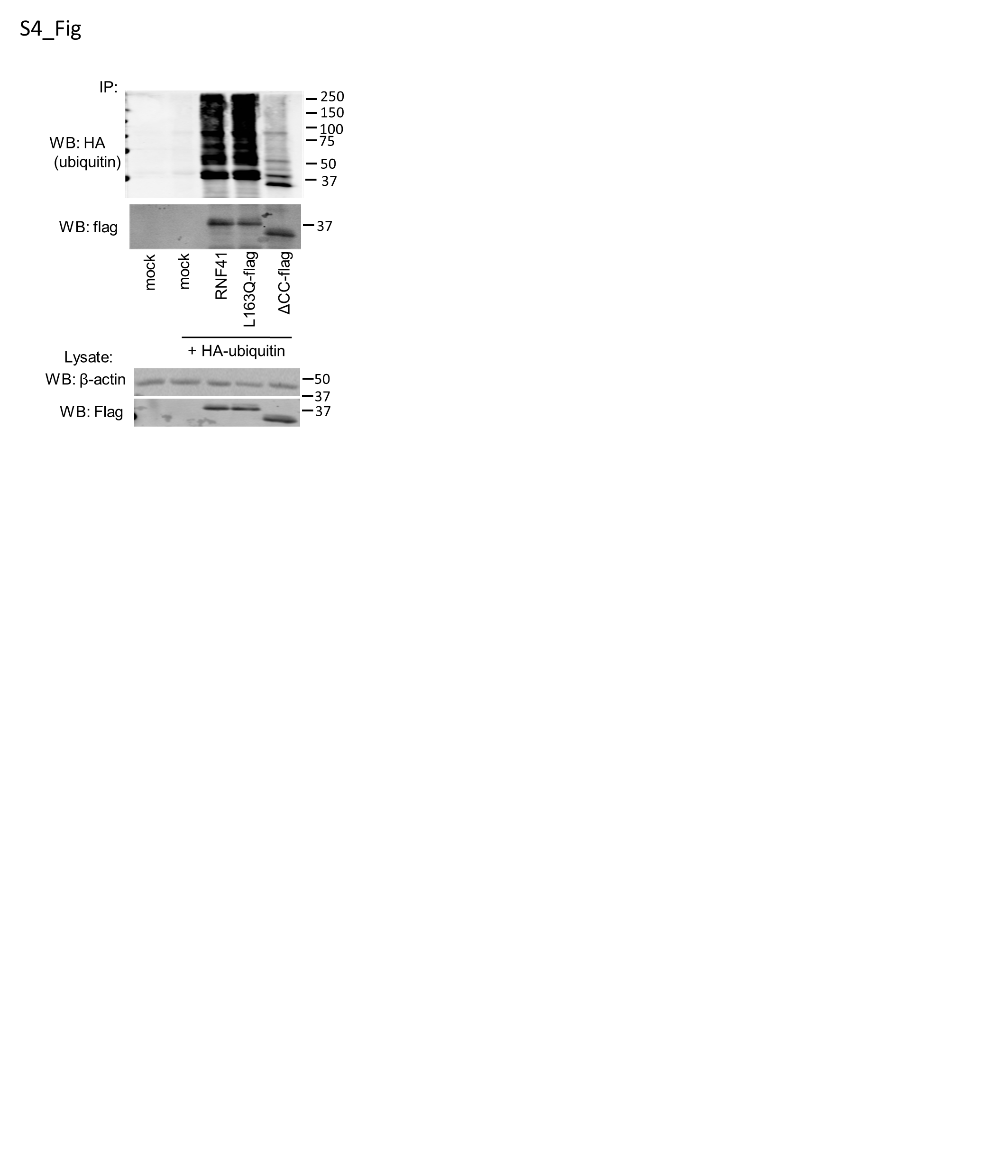

Supplement: S4 Fig — HEK293T cells co-transfected with pMet7-vectors encoding Flag-tagged RNF41, L163Q, ΔCC or sol IL5Rα (mock), together with HA-ubiquitin were incubated overnight with 5μM MG132 and 25μM chlolorquine to inhibit proteasomal or lysosomal degradation. Flag immunoprecipitation followed by anti-HA staining revealed the ubiquitination state of the RNF41 mutants. (TIF) [file pone.0178132.s007.tif]

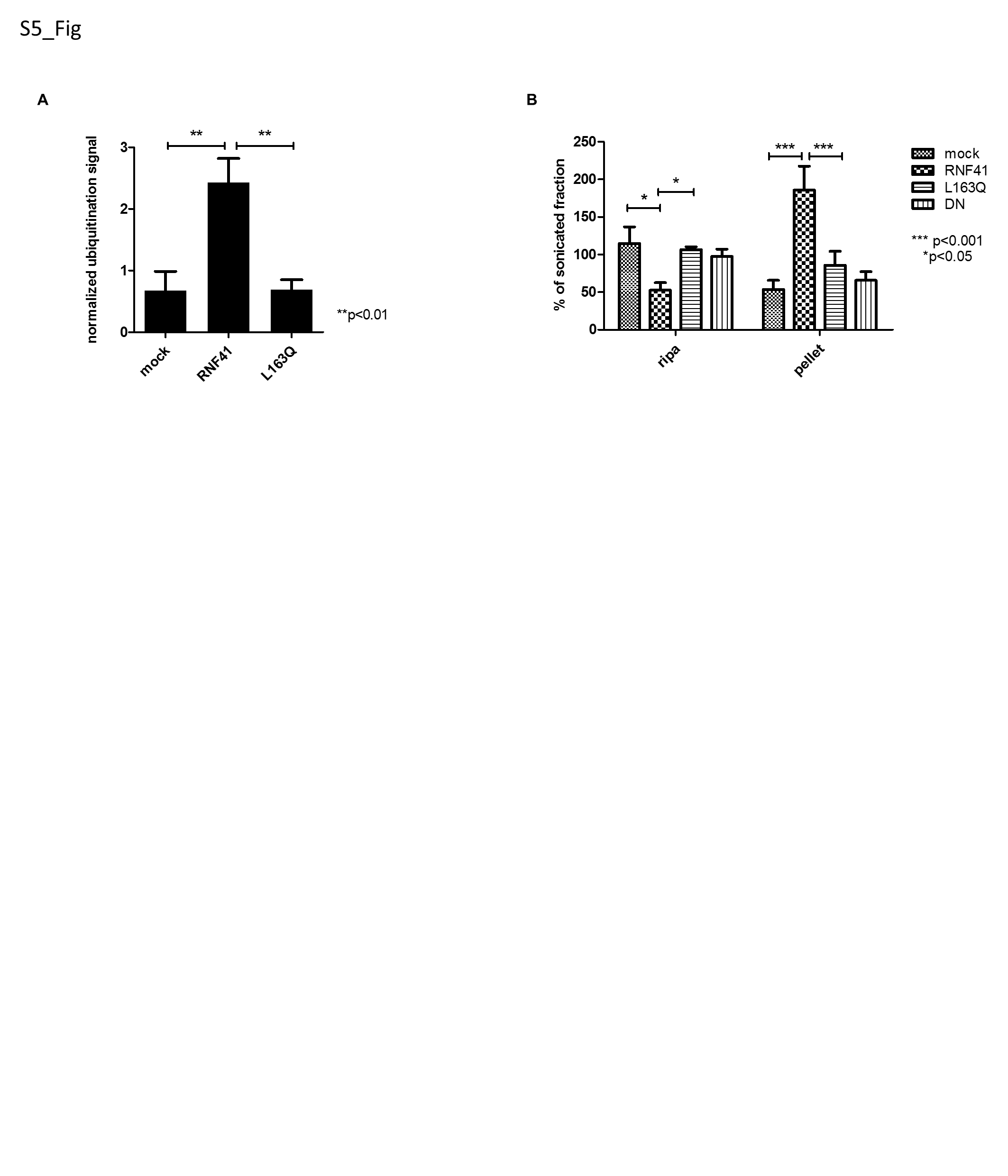

Supplement: S5 Fig — The Western Blots in Fig 4A and 4B, together with biological replicates, were quantified using Image J. (A) The ubiquitination signal was normalized for the amount of immunoprecipitated Flag-tagged VPS52 and compared between the mock, RNF41 and L163Q condition. A one-way ANOVA (randomized block design) showed a significant difference in VPS52 ubiquitination between the RNF41 and mock or L163Q transfected cells (p<0.01). (B) For each condition (i.e. mock, RNF41, L163Q and DN ectopic expression) the amount of VPS52 in the soluble RIPA and insoluble pellet fraction was compared to the total amount of VPS52 in the sonicated fraction. These results were subjected to a two-way ANOVA with post-hoc comparison (Bonferroni correction) that showed statistical difference between RNF41 and mock or L163Q (p<0.05) for the RIPA fraction and between RNF41 and mock or L163Q (p<0.001) for the pellet fraction. (TIF) [file pone.0178132.s008.tif]
